# Associations between weather conditions and osteoarthritis pain: a systematic review and meta-analysis

**DOI:** 10.1080/07853890.2023.2196439

**Published:** 2023-04-20

**Authors:** Lin Wang, Qinguang Xu, Yan Chen, Zhaohua Zhu, Yuelong Cao

**Affiliations:** a Research Institute of Orthopaedics & Traumatology, Shuguang Hospital affiliated to Shanghai University of Traditional Chinese Medicine, Shanghai, P.R. China; b Shanghai Municipal Hospital of Traditional Chinese Medicine, Shanghai University of Traditional Chinese Medicine, Shanghai, P.R. China; c Clinical Research Centre, Department of Orthopaedics, Zhujiang Hospital, Southern Medical University, Guangzhou, P.R. China

**Keywords:** Weather conditions, osteoarthritis pain, systematic review, meta-analysis

## Abstract

**Background:**

Although there is an assertion that weather conditions affect osteoarthritis (OA) pain, the results from clinical studies remain inconsistent. This meta-analysis was performed to evaluate the association between weather conditions and OA pain.

**Methods:**

Cochrane Library, Embase, PubMed, and Web of Science were searched from inception to September 30, 2022. Observational studies that explored all weather conditions associated with pain intensity were included. In the systematic review, the methodological quality of the selected studies was assessed and a best-evidence synthesis was used to make qualitative conclusions. Based on homogeneous results, Fisher’s *Z* scores derived from the effect size of temperature (T), barometric pressure (BP) or relative humidity (RH) related to OA pain were synthesized and further transformed to the correlation coefficients (summary r) in meta-analysis.

**Results:**

A total of 14 studies were included in the best-evidence synthesis of a qualitative systematic review. There was strong evidence with 13 of 14 studies reporting consistent findings that weather factors in general, including any kind of meteorological condition, were associated with OA pain. Subsequently, 3 studies regarding BP or T, and 5 studies regarding RH with the pain of OA were included in quantitative meta-analyses. Both BP (pooled Fisher’s *Z* = 0.37, 95% CI 0.15 to 0.59; summary *r* = 0.35, 95% CI 0.15 to 0.53) and RH (pooled Fisher’s *Z* = 0.10, 95% CI 0.01 to 0.18; summary *r* = 0.086, 95% CI −0.05 to 0.22) were positively related to OA pain, while T was negatively related to OA pain (pooled Fisher’s *Z* = −0.38, 95% CI −0.60 to −0.16; summary *r* = −0.36, 95% CI −0.54 to −0.16).

**Conclusions:**

In this study, weather factors in general were significantly associated with OA pain. It may provide useful references for the daily health management of OA. More studies designed with the consistent meteorological condition are warranted to validate the findings.

**KEY MESSAGE**
- Many people with osteoarthritis think their joint pain is affected by the weather, while the association between OA pain and weather conditions is still unclear.
- This is a systematic review and meta-analysis of 14 observational studies for the association between weather conditions and OA pain.
- Weather conditions appear to be associated with OA pain. Barometric pressure and relative humidity were positively correlated to OA pain intensity, while temperature was negatively correlated to OA pain.

## Introduction

Osteoarthritis (OA) is a multifactorial disease characterized by synovial inflammation, articular cartilage degeneration, and subchondral bone thickening [1–3], primarily leading to joint pain, stiffness, and disability [4,5]. Pain is the predominant symptom of OA, which usually leads to seek medical care. The signature pathologic feature of OA is articular cartilage loss. The mechanisms of pain in OA are still unclear. Although the signature pathologic feature of OA is articular cartilage loss, OA is widely recognized as a disease involving the whole joint including ligaments, menisci, synovitis and joint capsule [6]. Besides these factors, new bone formation, subchondral sclerosis, osteophytes, subchondral boundary cysts and bone marrow lesions (BMLs) are also significantly associated with OA [7]. Despite some intrinsic risk factors, such as gender, age and obesity [8,9], there is a widespread and frequent assertion that OA pain is influenced by the weather [10–17]. However, scientific studies have documented inconsistent findings. For example, Guedj revealed that 83% of arthritic patients were sensitive to changes in temperature (T), barometric pressure (BP), and precipitation (Pre), which may aggravate patient symptoms [18]. Mihye found that higher temperature and relative humidity (RH) could increase joint pain [19]. On the contrary, Timmermans concluded that low temperatures could reinforce joint pain of OA in older patients [20]. Two previous narrative reviews [21,22] without methodological quality evaluation have concluded an influence of weather on OA pain, but one recently published review showed that the correlation between BP, T, RH and pain was conflict [22]. The effect of climate conditions on OA was therefore ambiguous, it is necessary to perform meta-analysis to quantitatively synthesize the findings. Since human joints are usually exposed to the climate during their lifetime, exploring the effect of climate on OA may provide future benefits for research and clinical management.

Hence, the aim of this study was to synthesize up-to-date evidence on the association between weather parameters and OA pain. In this field, most of research designs were observational as it was hard to conduct randomized controlled trials (RCTs). We adopted a best-evidence synthesis method to systematically review those studies, and further applied a meta-analysis to those studies with homogenous effect size.

## Methods

Observational studies detecting the association between OA pain and climate conditions were searched for systematic review and meta-analysis. We reported this study following the Preferred Reporting Items for Systematic Reviews and Meta-Analyses Protocols (PRISMA) guideline [23] and Meta-analysis of Observational Studies in Epidemiology (MOOSE) reporting guideline [24]. The study protocol was registered with PROSPERO (CRD 42020223399) and has been published previously.

### Search strategy and selection criteria

Two researchers (LW and QX) undertook the research strategy collaboratively. Published studies in English were systematically searched from inception to 30 September 2022 in Cochrane Library, Embase, PubMed, and Web of Science. Medical Subject Headings (MeSH) and free text words related to “osteoarthritis”, “meteorological factors”, “climate”, and “weather” were used for searching. Ongoing or unpublished studies were searched manually in academic journals. The subject heading and keywords for the database are described in Supplement information (SI) 1.

The reviewers (LW and QX) were also responsible for selecting the appropriate studies in accordance with the inclusion criteria. In case both reviewers failed to achieve a consensus, a senior investigator (YC) intervened to resolve the situation.

We included all observational studies (cohort studies or case-crossover studies) focusing on relationships between different weather conditions and OA, with the exposure of any form of weather conditions (T, BP, RH, Pre, wind speed (WS), hours of sunshine (SH)) at any level associated with pain intensity. Animal studies, reviews, case reports, studies with an indefinite diagnosis of OA, and no detailed description of weather conditions were excluded.

Our primary outcome was the pain intensity of patients with OA. The effect size was the measure of the association between weather conditions and OA pain, including odds ratios (ORs), the Pearson(r), Spearman (Rs), unstandardized (B) and standardized (β) coefficients.

### Data extraction

Data from included studies were extracted into a standardized extraction form. Two authors (LWand QX) individually extracted the following data: country, study design, average age, diagnosis criteria, weather conditions including T, RH, BP, Pre, and WS, follow-up period, and outcomes assessments. Potentially suitable literatures could be further excluded due to incomplete data.

### Qualitative assessment

For the qualitative analysis, slightly modified criteria (SI 2) of prognostic factors for musculoskeletal disorders were used for methodological quality assessment and best-evidence synthesis [25,26]. The final criteria consisted of 17 items with each having a “±/?” answer option. A positive response for a certain question was scored one point, and an inconsistent or unclear response was scored zero points. The quality scores (maximum score of 16 points for a cohort study and 14 points for a case-crossover study) of each paper were added to calculate the overall internal validity. A high-quality study was defined if the overall internal validity score was ≥60%. Next, we performed a best-evidence synthesis. We first classified these studies according to the study design. A cohort study was thought to be one with a highly credible design, and followed by a case-crossover or case-control study. Then, we ranked the studies according to the overall internal validity score and further formulated the levels of evidence. Strong evidence of weather-influencing OA was considered when 3 or more high-quality cohort studies and ≥75% of the studies reported consistent findings were provided. Moderate evidence was considered to be with 2 high-quality cohort studies, 2 or more high quality case-crossover studies, 3 or more high-quality case-crossover studies were provided. Limited evidence was considered to be with a single cohort study, one or two case-crossover studies, or multiple case-crossover studies. Conflicting evidence was defined by conflicting findings (i.e. <75% of the studies reported consistent findings).

### Statistical analysis

For meta-analysis, Review Manager 5.2 was used to pool Fisher’s Z or OR with 95% CI. Fisher’s Z was transformed from the r, Rs, B and β coefficients of each weather condition by the following steps. Firstly, B was converted to β, so that β or Rs could be converted into r using related formulas [27,28]. Then, the r was converted to Fisher’s *Z* values (z sez) for the final meta-analysis [29–31]. The overall effect size for each pooled analysis was calculated by the weighted mean of the inverse variance-adjusted individual Fisher’s *Z*. Stata 15 was used to transform the overall Fisher’s Z to the correlation coefficients (summary r) assessing the relationship between weather and OA pain. All conversion formulas and relevant codes were listed in SI 3 and SI 4 in the Supplement. As suggested by Cohen’s scientific definition of determining relationships, summary *r* = 0.1 − 0.3 is a weak correlation, summary *r* = 0.3 − 0.5 is a moderate correlation, and summary *r* = 0.5 − 1.0 is a strong correlation. A random-effects model (REM) was performed when high heterogeneity existed. Otherwise, the fixed effects model (FEM) was used. High heterogeneity was generally defined as the standard χ^2^ value with a significance level of *p* < 0.10, *I*^2^ > 50%.

## Results

### Search results and study characteristics

**Figure 1.**
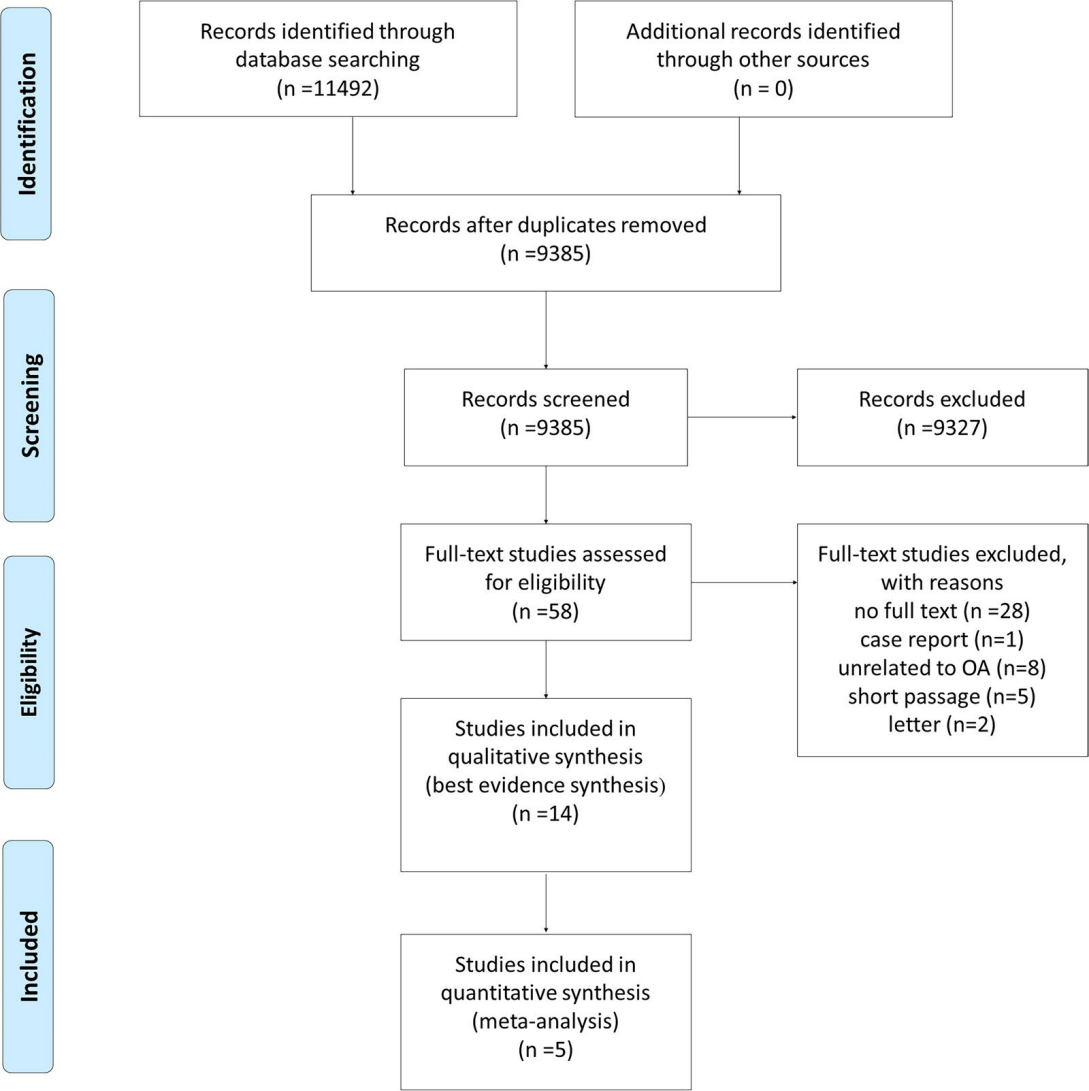
Preferred reporting items for systematic reviews and meta-analyses (PRISMA) flow diagram of search results.

The study flow diagram outlined the study selection and reasons for exclusion (Figure 1). A total of 11492 original reports were searched from PubMed (*n* = 2725), Embase (*n* = 3553), Cochrane (*n* = 520), and Web of Science (*n* = 4694). From 9385 studies screened for title and abstracts, 58 studies were selected for full-text examination. In this procedure, 44 studies were excluded for below reasons: no full text (*n* = 28), case report (*n* = 1), letter (*n* = 2), unrelated to OA (*n* = 8), short passages (*n* = 5). Finally, 14 studies were included in the best-evidence synthesis.

Of those, 9 studies reported Rs [32,33], r [17,34], B [20,35] or β [36–38] values. In the end, 5 studies were included for synthesizing the transformed Fisher’s Z in meta-analysis. The reasons for the exclusion of other literatures were as follows: no standard deviation (SD) of weather variables [20], inconsistent weather variables [35,38], and unclear sample size [33].

Three studies reported OR and 95% CI values. After excluding one study due to inconsistent weather variables [39], we were unable to synthesize OR in meta-analysis due to insufficient study numbers (*n* = 2) [40,41].

Collectively, 6 meteorological factors (T, RH, BP, WS, Pre, and SH) were concluded in this work. A total of 2102 OA patients from more than 10 countries were included, 511 cases were performed in meta-analysis for 3 meteorological factors (T, RH and BP). Pain intensity assessments for OA were the numeric rating scale of pain (NRS) for 4 studies [17,20,40,41], visual analogue scale (VAS) for 7 studies [32–35,38,39,42], the Western Ontario and McMaster University osteoarthritis index (WOMAC) for 2 studies [37,43], and a pain questionnaire (0: no pain, 1: pain, 2: extreme pain) for 1 study [18]. Twelve studies used a prospective cohort study design, and case-crossover study was designed in 2 studies. Mean age varied from 60 years to 73.8 years. The follow-up periods ranged from 1 month to 2 years. OA joints in this study included hip, knee, foot, and shoulder. Knee [32,35,37,41] and hip [36,38,40] OA were more frequently reported. Individual characteristics of included studies were detailed in Table 1.

**Table 1.** Characteristics of 14 studies.

Author (year country)	Study design	Disease	Age	Exposure	Follow up periods	Participants	Pain assessment	Effect size
Guedj D [18] 1990 Israel	CH	OA	61	T(°C) Pre(mm) RH(%) BP(hPa)	1 month	24	Pain questionnaire 0: no pain, 1: pain, 2: extreme pain	Prediction rate of pain T BP Pre: 57.6%
Queiroga [42] 2013 Brazil	CH	Hand OA	62 ± 8.8	T(°C) RH(%) BP(hPa) Pre(mm)	2 months	32	VAS (0 to 10)	R^2^ T, BP 52 to 88%
Timmermans EJ [20] 2015 European	CH	OA	73.8 ± 5	RH(%) T(°C) Pre(mm) WS(m/s) BP(hPa)	24 months	810	NRS (0 to 10) increase of ≥2 points	B (95%CI) daily average: RH 0.005 (0.002 to 0.008), Pre −0.006(–0.012 to–0.001), WS −0.012(–0.040 to −0.017), 3–day average: RH 0.004 (–0.001 to 0.007), BP −0.003 (–0.008, −0.002)
Peultier L [35] 2016 French	CH	Knee OA	65 ± 9	T(°C) RH(%) Pre(mm) BP(hPa) SH(h) WS(m/s)	9 months	113	VAS (0 to 100)	B MaT 0.298, Min RH 0.238
Dorleijn DMJ [36] 2014 Netherlands	CH	Hip OA	63.4 ± 9	RH(%) Pre(mm) BP(hPa) SH(24 h)T(°C) WS(m/s)	24 months	222	WOMAC Pain (0 to 100) increased by 1.0 scale	β (95%CI) RH 0.1(0.0 to 0.2)
Brennan SA [38] 2012 Ireland	CH	Hip OA	NA	BP(0.75 hPa) Pre(mm) T(°C)	1 month	53	VAS (0 to 10)	β BP −0.024
McAlindon T [37]2007 the United States	CH	Knee OA	60 ± 9.4	T(°C) BP(10 hPa) Pre(25.4 mm) RH(%)	3 months	200	WOMAC Pain (0 to 100)	β BP −0.001, T −0.01, Pre −0.5, RH 0.001, Dew point −0.01
Vergés J [39] 2004 Spain	CH	OA	NA	T(°C) BP(hPa) RH(%)	1 month	80	VAS (0 to 100)	OR (95%CI) T 1.042(0.854 to 1.27), BP 0.793(0.647 to 0.972), RH 0.963(0.784 to 1.183)
Fu K [40] 2020 Australian	CC	Hip OA	62.9 ± 8	T(°C) BP(hPa) Pre(mm) RH(%)	3 months	129	NRS (0 to 10) increase of ≥ 2 points	0R (95%CI) MaT < 15 0.82(0.44 to 1.56), MaT 25–35 0.94(0.57 to 1.54) MaT > 35 1.6(0.59 to 4.34), MiT < 10 0.97(0.63 to 1.51) MiT > 20 0.68(0.35 to 1.36), TV 10–20 1.12(0.78 to 1.6) TV > 20 3.89(1.04 to 14.48), RH < 59 1.17(0.84 to 1.65) RH > 79 0.68(0.4 to 1.14), Pre 0–5 0.96(0.61 to 1.52) Pre > 10 0.62(0.28 to 1.34), BP < 1010 0.84(0.5 to 1.43) BP 1010–1014 1.16(0.78 to 1.73), BP 1019–1024 1.04(0.67 to 1.63), BP > 1024 1.03(0.6 to 1.9)
Ferreira ML [41] 2016 Australia	CC	Knee OA	61.7 ± 8.7	Pre(mm) T(°C) RH(%) BP(hPa)	3 months	171	NRS (0 to 10) increase of ≥ 2 points	OR (95%CI) MaT < 10 0.58(0.03 to 10.25), MaT 20–30 0.96(0.68 to 1.36), MaT >30 2.18(1.01 to 4.74), MiT < 10 1.01(0.78 to 1.54), MiT 20–30 1.91(0.73 to 4.95), RH < 59 1.02(0.73 to 1.42), RH > 79 1.25(0.72 to 2.19), Pre 0–5 1.16(0.73 to 1.82), Pre > 10 1.35(0.66 to 2.78), BP < 1010 0.89(0.51 to 1.54), BP 1010–1014 1.38(0.93 to 2.06), BP 1019–1024 1.41(0.94 to 2.12), BP > 1024 0.83(0.44 to 1.58)
Strusberg I [34] 2002 Argentina	CH	OA	65.85 ± 9.70	T(°C) RH(%) BP(hPa)	12 months	52	VAS (0 to 10)	r T −0.38, BP 0.37, RH 0.24
Ziadé N [17] 2021 Lebanon	CH	OA	63.6 ± 10.3	T(°C) RH(%) BP(hPa)	12 months	27	NRS (0 to 10)	r T −0.35, RH −0.323, BP 0.384
Wilder FV [33] 2003 the United States	CH	OA(neck, hand, shoulder, knee and foot)	72	BP(hPa) Pre(10 mm) T(°C)	19-23 months	154	VAS (0 to 10)	Rs MT 0.08, MiT 0.07, MaT 0.08, BP 0.02, Pre 0.06, MT1–day lagging 0.07 MT 1–day leading 0.07, BP 1–day lagging 0.04, BP 1–day leading 0.10 Pre 1–day lagging 0.00, Pre 1–day leading 0.02
Cay HF [32] 2009 Turkey	CH	Knee OA	63.6 ± 6.8	BP(hPa) T(°C) SH(h) Pre(mm) RH(%) WS(m/s)	8 months	10	VAS (0 to 10)	Rs T −0.282, RH 0.15, SH −0.242, BP 0.13, WS 0.02, Pre 0.109, Evaporation −0.232

CH: cohort study; CC: case-crossover study; T: temperature; BP: barometric pressure; RH: relative humidity; WS: wind speed; SH: hours of sunshine; Pre: precipitation; TV: temperature variation; MT: mean temperature; NRS: numeric rating scale of pain; VAS: visual analogue scale; WOMAC: Western Ontario and McMaster University osteoarthritis index; NA: not applicable; r: correlation coefficient; Rs_:_ spearman correlation coefficients; OR: odds ratio; B: unstandardized coefficient; β: standardized coefficient; R^2^: multiple regression; 1-day lagging/leading = weather index value 1 day prior/subsequent to the pain assessment.

### Quality assessment of literatures

In this work, 14 studies including 11 high-quality studies, 2 high-quality case-crossover studies and 1 low-quality study were evaluated. The internal validity score of all studies ranged from 56% to 93% [39,41], and most studies scored from 63% to 75% [17,18,20,32–35,37,42]. In these studies, main scores contributed in description of baseline data, prospective study design, and reasonable and rigorous statistical methods. The loss of points was mainly concentrated in short follow-up time, rough and incomplete information on completers vs withdrawals, and absence of OA assessing reproducibly, which were presented in SI 5.

### Best-evidence synthesis for the overall relationship between weather conditions and OA pain

As for best-evidence synthesis, all weather conditions like T, RH, Pre and BP were grouped to explore the association with OA. In this work, 13/14 of the studies reported consistent findings that OA pain was influenced by meteorological factors (Table 2). Among these studies, T reported a significant association with OA pain in 8 studies, BP was reported in 8 studies, RH was reported in 7 studies, Pre was reported in 2 studies, SH was reported in 1 study. T, RH and BP were the most frequent weather factors related to OA pain as reported.

**Table 2.** Best evidence synthesis of association between overall weather types and OA pain.

Author year	Design	Exposure*	Evidence synthesis level	
Vergés J [39] 2004	CH	BP	Strong^#^	
Guedj D [18] 1990	CH	T, Pre,BP		
McAlindon T [37] 2007	CH	T, BP		
Brennan SA [38] 2012	CH	BP		
Ziadé N [17] 2021	CH	T, BP,RH		
Timmermans EJ [20] 2015	CH	RH		
Queiroga [42] 2013	CH	T, RH		
Wilder FV [33] 2003	CH	BP		
Peultier L [35] 2016	CH	T, RH		
Strusberg I [34] 2002	CH	T, BP,RH		
Cay HF [32] 2009	CH	T, BP,Pre,RH,SH		
Dorleijn DMJ [36] 2014	CH	RH,BP		
Fu K [40] 2020	CC	T		
Ferreira ML [41] 2016	CC	NA		

CH: cohort study; CC: case-crossover study; T: temperature; BP: barometric pressure; RH: relative humidity; SH: hours of sunshine; Pre: precipitation; *: any one or more statistically significant weather factors related to OA pain as reported; NA: not applicable; #: strong evidence level generalized when 3 or more high-quality cohort studies and ≥75% of the studies reported consistent findings.

Considering whether the weather factors have different effects on OA pain in small joints or large joints, we performed a stratified qualitative summary. There was strong evidence that knee OA pain was influenced by meteorological factors as 3/4 of high-quality studies reported consistent findings. T, RH and BP were the most frequent weather factors related to knee OA pain. There was moderate evidence that hip OA pain was influenced by meteorological factors, especially BP. While one high-quality cohort study reported that hand OA pain was influenced by T and RH, the evidence synthesis level is limited (SI 6).

### Meta-analysis

**Figure 2.**
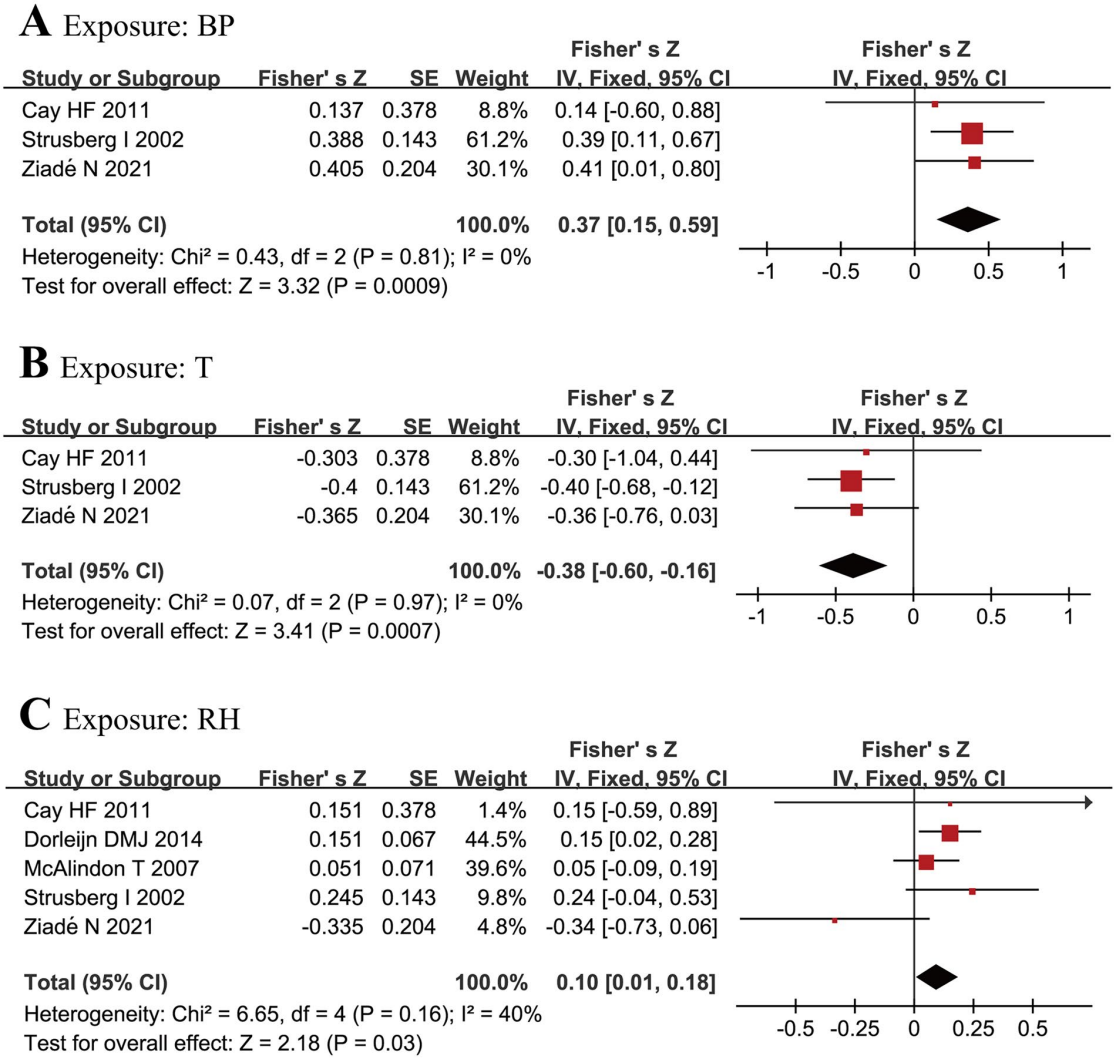
Forest plot of Fisher’s Z between OA pain and BP RH T.

The results showed that weather factor of T was negatively correlated to OA pain, while BP and RH were positively correlated to OA pain. Four studies [15,30,32,37] were initially included in the meta-analysis of the relation between BP, T and OA pain, but one of them [37] had strong heterogeneity (*I*^2^ =63.9%, *p* = 0.04 for heterogeneity in BP group, or *I*^2^ =61.9%, *p* = 0.049 for heterogeneity in T group). After excluding this study, BP was positively related to OA pain with a pooled Fisher’s *Z* 0.37 (95% CI 0.15 to 0.59), and there was no heterogeneity among the studies (*I*^2^ = 0.0%, *p* = 0.81). T was negatively impacted on OA pain with a pooled Fisher’s Z −0.38 (95% CI −0.60 to −0.16), with no heterogeneity among the 3 studies (*I*^2^ = 0.0%, *p* = 0.97) (Figure 2(A,B)). There were 5 studies [15,30,32,34,37] included in the meta-analysis of the relationship between RH and OA pain. RH was positively related to the pain with a pooled Fisher’s Z 0.1 (95% CI 0.01 to 0.18), with no obvious heterogeneity (*I*^2^ =40%, *p* = 0.16) among 5 studies (Figure 2(C)).

We further converted the pooled Fisher’s *Z* into the summary r to account for the relationship between the 3 types of meteorological factors and OA pain. BP (summary *r* = 0.35, 95% CI 0.15 to 0.53) and T (summary *r* = −0.36, 95% CI −0.54 to −0.16) had moderate correlation, while RH (summary *r* = 0.086, 95% CI −0.05 to 0.22) had weak correlation (Table 3).

**Table 3.** Meta analysis results of Fisher’s Z and the summary r between BP, RH, T and OA pain.

Weather conditions	Tests of overall effects of Meta-analysis				Summary	
	*Z*	*P*	Fisher’s Z	95%CI	*r*	95% CI
BP	3.32	<0.001	0.37	0.15 to 0.59	0.35	0.150 to 0.53
RH	2.18	0.030	0.10	0.01 to 0.18	0.086	–0.05 to 0.22
T	–3.41	<0.001	–0.38	–0.6 to −0.16	–0.36	–0.54 to −0.16

BP: barometric pressure; RH: relative humidity; T: temperature; r: transformed by the pooled Fisher’s *Z*

### Publication bias

**Figure 3.**
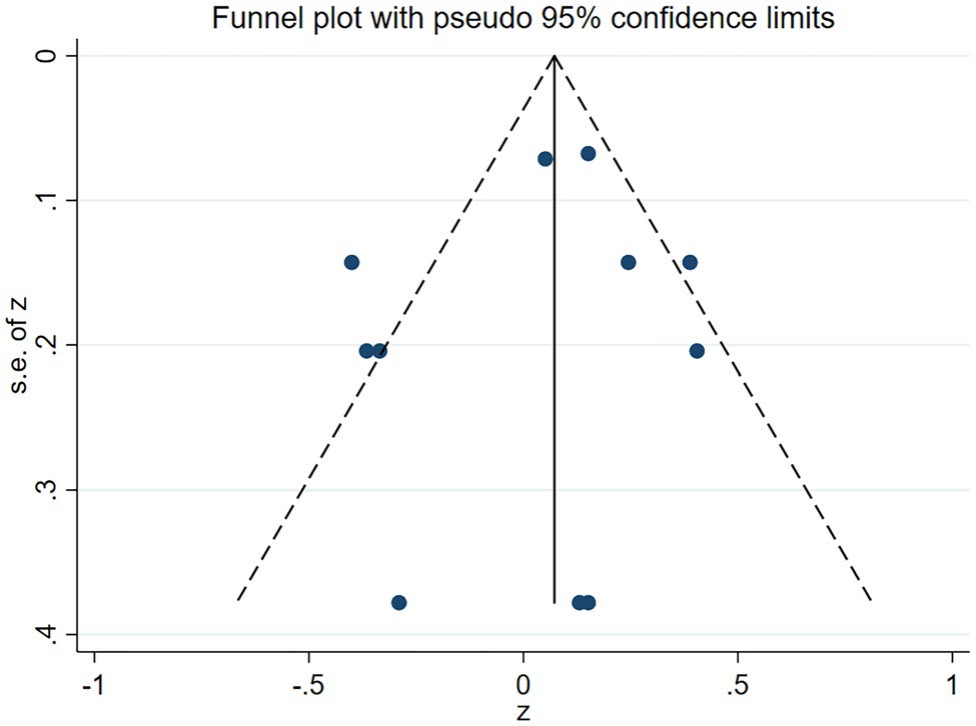
Funnel plot of weather variables.

Across all weather variables, there was a low risk of selection bias by visual inspection of the funnel plot (Figure 3).

## Discussion

To the best of our knowledge, this was the first study to investigate the association between weather conditions and OA pain through qualitative and quantitative analysis. Fourteen studies were synthesized in our systematic review. Based on our synthesis of evidence, strong evidence was found that weather conditions in general, including any meteorological condition, were associated with OA pain. As qualitative analysis was difficult to identify the relational degree or direction between certain type of weather factor and OA pain, further meta-analysis was performed to determine whether the correlation was positive or negative. Overall, weather factors were related to OA pain severity. More well-designed studies, using standardized reference of climate conditions, are required to refine the results.

Our finding may have broad applicability to those OA patients who are more vulnerable to weather conditions. An early European study reported that 67.2% of participants with OA attributed their pain to the weather [15]. Evidence from recent study also demonstrated the association of weather sensitivity and clinical symptoms and structural degradations in knee OA patients. 57.5% of weather-sensitive individuals more likely had severe knee pain, dysfunction and as well as cartilage defects [16]. When clinical research evidence increasingly demonstrates the aggravation of weather conditions such as T, RH and BP on OA pain, weather factors should be considered in OA management. Our finding may also contribute to the development of basic research on weather impacting OA pain. The function of Thermosensitive Transient Receptor Potential channels (Thermo-TRPs) was related with the weather stimulating, which may be the possible pathophysiological mechanisms of OA pain [44–47]. TRPs are a large family of proteins including 6 main subfamilies termed the TRPC (canonical), TRPV (vanilloid), TRPM (melastatin), TRPP (polycystin), TRPML (mucolipin), and TRPA (ankyrin) groups [48]. Andersson reported the TRPA-1 was overexpressed after exposure to cold temperatures (10 °C), and the mice demonstrated more nocifensive behaviour and mechanical pain sensitivity [49,50]. These quantitative analysis findings, positive or negative effect of BP, RH, and T on OA pain, may provide the basic theories and help to set weather parameters for future studies exploring these potential mechanisms.

Our study has some strengths. This study combined qualitative and quantitative research methods which not only made a qualitative judgment of the correlation between weather factors and OA, but also detected the degree of positive or negative coefficient of specific weather conditions on OA. Participants from multiple countries and regions with multiple sites of OA (neck, hand, shoulder, knee, hip and foot) were included in this systematic review.

This study also has limitations. Firstly, included literatures were all observational studies which may have potential confounding bias compared with RCTs. However, it is difficult to use RCT design in the real world in the study of weather as the observation factor which is completely out of people’s subjective control. Interestingly, a previous double-blind study was performed by Hollander and Yeostros where 4 OA patients were placed in a climatized room for 2 weeks with controlled BP, T, and RH [10]. The result showed that the simultaneous variation of the RH and pressure affected arthritis symptoms. This conclusion also supported our results. Due to the reported symptom assessment that did not give clear status on pain evaluation, this laboratory study was excluded from our systematic review. Second, there were diverse expression of effect size of the study and different classification of specific weather conditions applied in literatures. This makes the number of literatures eventually included in the meta-analysis relatively small. To be specific, some studies reported OR values and some reported correlation coefficient values, which were difficult to combine for effect size. On the other hand, two studies [35,38] that were eligible for synthesizing effect sizes were eventually excluded due to inconsistent weather variables. Moreover, studies in our research were carried out in different countries, which have various climates and weather conditions. However, these weather conditions were measured in the same unit with similar range of variation that may reduce the influence of meteorological variables on the result. Also, we had to transform β or Rs for summary r. Nevertheless, the studies included in our meta-analysis showed good homogeneity. Therefore, further studies with standardized weather conditions and effect sizes are needed. Thirdly, the result may be biased in terms of outcome measures. Although we adopted consistent assessment tool of VAS, OA pain is a highly subjective symptom and can be influenced by many factors, such as the subjective questionnaire used to evaluate pain intensity may be influenced by confounding factors from different culture beliefs, educational level, tolerance, expressiveness and even psychological status [16,51–53]. In addition, the study population was mostly older people, who tend to be less resistant to bad weather than younger people.

## Conclusions

In this systematic review and meta-analysis, weather conditions appear to be associated with OA pain. For the specific meteorological condition, quantitative analysis showed the moderate correlation between OA pain and T or BP, with RH bearing weak correlation. More well-designed studies, using standardized weather conditions and effect sizes are warranted to validate the influence of weather factors on OA.

## Supplementary Material

Supplemental Material

Supplemental Material

Supplemental Material

Supplemental Material

Supplemental Material

Supplemental Material

## Data Availability

All data generated or analysed during this study are included in this published article and its supplementary information files. The data synthesized and presented in the results section have been well-referenced as an update systematic review article. However, raw data (in excel sheet) used in the statistical analysis will be made available on request through the corresponding author (Yuelong Cao).
