# Supplementary material for: Associations between weather conditions and osteoarthritis pain: a systematic review and meta-analysis
Source: Ann Med. 2023 Apr 20;55(1):2196439. doi: 10.1080/07853890.2023.2196439 (PMC10120534; doi:10.1080/07853890.2023.2196439)
Supplement: Supplemental Material [file IANN_A_2196439_SM6340.docx]

SI 6 Best evidence synthesis of association between overall weather types and knee, hip, hand OA pain

(1) Best evidence synthesis of association between overall weather types and knee OA pain

| Location | Author year | Design | Exposure* | Evidence Synthesis Level | |
| --- | --- | --- | --- | --- | --- |
| Knee OA | McAlindon T^[37]^ 2007 | CH | T、BP | Strong# |  |
|  | Peultier L^[35]^ 2016 | CH | T、RH |  |  |
|  | Cay HF^[32]^ 2009 | CH | T 、BP、Pre、RH、SH |  |  |
|  | Ferreira ML^[41]^ 2016 | CC | NA |  |  |

CH: cohort study, CC: case-crossover study, T: temperature, BP: barometric pressure, RH: relative humidity, SH: hours of sunshine, Pre: precipitation, *: any one or more statistically significant weather factors related to OA pain as reported, NA: not applicable.

# 3 high-quality cohort studies and 3/4 of studies reported consistent findings that knee OA pain was influenced by meteorological factors. Of these, T was reported in 3 studies, BP and RH were reported in 2 studies, and Pre and SH were reported in 1 study. T, RH and BP were the most frequent weather factors related to knee OA pain.

(2) Best evidence synthesis of association between overall weather types and hip OA pain

| Location | Author year | Design | Exposure* | Evidence Synthesis Level | |
| --- | --- | --- | --- | --- | --- |
| Hip OA | Brennan SA^[38]^ 2012 | CH | BP | Moderate# |  |
|  | Dorleijn DMJ^[36]^ 2014 | CH | RH、BP |  |  |
|  | Fu K^[40]^ 2020 | CC | T |  |  |

CH: cohort study, CC: case-crossover study, T: temperature, BP: barometric pressure, RH: relative humidity. *: any one or more statistically significant weather factors related to OA pain as reported.

# 2 high-quality cohort studies reported consistent findings that knee OA pain was influenced by meteorological factors. Among the factors, T and RH were reported in 1 study, and BP was reported in 2 studies. BP was the most frequent weather factor related to hip OA pain.

(3) Best evidence synthesis of association between overall weather types and hand OA pain

| Location | Author year | Design | Exposure* | Evidence Synthesis Level | |
| --- | --- | --- | --- | --- | --- |
| Hand OA | Queiroga^[42]^ 2013 | CH | T、RH | Limited# |  |

CH: cohort study, T: temperature, RH: relative humidity, *: any one or more statistically significant weather factors related to OA pain as reported.

# 1 high-quality study reported that hand OA pain was influenced by T and RH, thus the result of evidence synthesis level is limited.
