# Supplementary material for: Associations between weather conditions and osteoarthritis pain: a systematic review and meta-analysis
Source: Ann Med. 2023 Apr 20;55(1):2196439. doi: 10.1080/07853890.2023.2196439 (PMC10120534; doi:10.1080/07853890.2023.2196439)
Supplement: Supplemental Material [file IANN_A_2196439_SM6339.docx]

SI 5 Results of literatures quality assessment

| Study | Design | DSP | DRC | SD | SDC | EAB | EMI | EA | OAI | PR | PVD | PD | FT | W | I | FIO | AAT | AMA | S | TS | QS |
| --- | --- | --- | --- | --- | --- | --- | --- | --- | --- | --- | --- | --- | --- | --- | --- | --- | --- | --- | --- | --- | --- |
| Vergés J^[37]^ 2004 | Cohort study | 1 | 0 | 1 | 0 | 1 | Na | 1 | 0 | N | 0 | 1 | 0 | 1 | 0 | 1 | 1 | 1 | 9 | 16 | 56 |
| Guedj D^[16]^ 1990 | Cohort study | 1 | 0 | 0 | 0 | 1 | Na | 1 | 1 | N | 1 | 1 | 0 | 1 | 0 | 1 | 1 | 1 | 10 | 16 | 63 |
| McAlindon T^[35]^ 2007 | Cohort study | 1 | 0 | 0 | 0 | 1 | Na | 1 | 1 | N | 1 | 1 | 0 | 1 | 0 | 1 | 1 | 1 | 10 | 16 | 63 |
| Brennan SA^[36]^ 2012 | Cohort study | 1 | 0 | 1 | 0 | 1 | Na | 1 | 1 | N | 1 | 1 | 0 | 1 | 0 | 1 | 1 | 1 | 11 | 16 | 69 |
| Ziadé N^[15]^ 2021 | Cohort study | 1 | 1 | 1 | 1 | 1 | Na | 1 | 1 | N | 1 | 1 | 0 | N | N | 1 | 1 | 1 | 11 | 16 | 69 |
| Timmermans EJ^[18]^ 2015 | Cohort study | 1 | 0 | 1 | 1 | 1 | Na | 1 | 1 | N | 1 | 1 | 0 | 1 | 0 | 1 | 1 | 1 | 12 | 16 | 75 |
| Queiroga^[40]^ 2013 | Cohort study | 1 | 1 | 1 | 0 | 1 | Na | 1 | 1 | N | 1 | 1 | 0 | 1 | 0 | 1 | 1 | 1 | 12 | 16 | 75 |
| Wilder FV^[31]^ 2003 | Cohort study | 1 | 0 | 1 | 0 | 1 | Na | 1 | 1 | N | 1 | 1 | 1 | 1 | 0 | 1 | 1 | 1 | 12 | 16 | 75 |
| Peultier L^[33]^ 2016 | Cohort study | 1 | 1 | 1 | 0 | 1 | Na | 1 | 1 | N | 1 | 1 | 0 | 1 | 0 | 1 | 1 | 1 | 12 | 16 | 75 |
| Strusberg I^[32]^ 2002 | Cohort study | 1 | 1 | 1 | 1 | 1 | Na | 1 | 1 | N | 1 | 1 | 0 | 1 | 0 | 1 | 1 | 1 | 12 | 16 | 75 |
| Cay HF^[30]^ 2009 | Cohort study | 1 | 1 | 1 | 1 | 1 | Na | 1 | 1 | N | 1 | 1 | 0 | 0 | 1 | 1 | 1 | 1 | 12 | 16 | 75 |
| Dorleijn DMJ^[34]^ 2014 | Cohort study | 1 | 1 | 1 | 1 | 1 | Na | 1 | 1 | N | 1 | 1 | 0 | 1 | 0 | 1 | 1 | 1 | 13 | 16 | 81 |
| Fu K^[38]^ 2020 | Case-crossover | 1 | 1 | 1 | 1 | 1 | 0 | 1 | 1 | 0 | 1 | 1 | NA | NA | NA | 1 | 1 | 1 | 12 | 14 | 86 |
| Ferreira ML^[39]^ 2016 | Case-crossover | 1 | 1 | 1 | 1 | 1 | 1 | 1 | 1 | 0 | 1 | 1 | NA | NA | NA | 1 | 1 | 1 | 13 | 14 | 93 |

N: unclear, NA: not applicable, DSP: Description of source population, DRC: Description of relevant inclusion and exclusion criteria, SD: Selection before disease was present or at uniform point, SDC: Sufficient description of baseline characteristics, EAB: Exposure assessment was blinded, EMI: Exposure was measured identically for cases and controls, EA: Exposure was assessed prior to the outcome, OAI: OA was assessed identical in studied population, PR: Presence of OA was assessed reproducibly, PVD: Presence of OA was according to valid definitions, PD: Prospective design was used, FT: Follow-up time ≥3 yr, W: Withdrawals≤20% , I: Information on completers vs withdrawals, FIO: Frequency of most important outcomes were given, AAT: Appropriate analysis techniques were used, AMA: Appropriate multivariate analysis techniques, S: Score, TS: Total score, QS: Quality score %
