# Supplementary material for: Associations between weather conditions and osteoarthritis pain: a systematic review and meta-analysis
Source: Ann Med. 2023 Apr 20;55(1):2196439. doi: 10.1080/07853890.2023.2196439 (PMC10120534; doi:10.1080/07853890.2023.2196439)
Supplement: Supplemental Material [file IANN_A_2196439_SM6336.docx]

**Installed codes**

findit metan

findit metaan

findit metafunnel

findit metabias6

findit midis

findit metaninf

findit metainf

**Analyze codes of Fisher’s Z values (z sez) transformed by r**

generate z = atanh(r)

generate sez= sqrt(1/(n-3))

**Analyze codes of summary r transformed by the overall Fisher’s Z from meta-analyses**

metan z sez, random second(fixed) label(namevar=Study) effect (Fisher’s Z)

display _newline "Pooled estimate of r = " tanh(r(ES))

display _newline "Lower Limit of 95% CI = " tanh(r(ci_low))

display _newline "Upper Limit of 95% CI = " tanh(r(ci_upp))
