# Supplementary material for: Associations between weather conditions and osteoarthritis pain: a systematic review and meta-analysis
Source: Ann Med. 2023 Apr 20;55(1):2196439. doi: 10.1080/07853890.2023.2196439 (PMC10120534; doi:10.1080/07853890.2023.2196439)
Supplement: Supplemental Material [file IANN_A_2196439_SM6329.docx]

**Conversion formula of B to β**

β=B*Sx/Sy

Sx: SD of weather,

Sy: SD of OA pain,

B: unstandardized coefficient,

β: standardized coefficient,

**Conversion formula of β to r**

r=β＋0.05λ

r: correlation coefficient

λ is an indicator variable that equals 1 when β is nonnegative and 0 when β is negative

**Conversion formula of Rs to r**

r= 2sin(Rs*π/6)

π=3.141593

Rs: Spearman coefficient.

**Fisher’s Z conversion formula of r to Z and Sez**

Z=0.5*ln((1+r)/(1-r))

Vz=1/(n-3)

Sez=Vz^0.5

N: sample of each study,

Vz: variance of z,

Sez: standard error of z.

**Conversion formula of the pooled Fisher’s Z to Summary r**

Summary r=(e^2z-1)/ (e^2z+1)

z: the pooled Fisher’s Z by meta-analysis of each weather variable.
