# Supplementary material for: Associations between weather conditions and osteoarthritis pain: a systematic review and meta-analysis
Source: Ann Med. 2023 Apr 20;55(1):2196439. doi: 10.1080/07853890.2023.2196439 (PMC10120534; doi:10.1080/07853890.2023.2196439)
Supplement: Supplemental Material [file IANN_A_2196439_SM6325.docx]

AF 2 Criteria list for the methodological assessment of studies

| Item | Criterion | CC/ CH | Score |
| --- | --- | --- | --- |
| Study population |  |  |  |
| 1 | Description of source population | CC/ CH | +/-/? |
| 2 | Description of relevant inclusion and exclusion criteria | CC/ CH | +/-/? |
| 3 | Selection before disease was present or at uniform point | CC/ CH | +/-/? |
| 4 | Sufficient description of baseline characteristics | CC/ CH | +/-/? |
| Assessment of risk factor |  |  |  |
| 5 | Exposure assessment was blinded | CC/ CH | +/-/? |
| 6 | Exposure was measured identically for cases and controls | CC | +/-/? |
| 7 | Exposure was assessed prior to the outcome | CC/ CH | +/-/? |
| Assessment of OA |  |  |  |
| 8 | OA was assessed identical in studied population | CC/ CH | +/-/? |
| 9 | Presence of OA was assessed reproducibly | CC/ CH | +/-/? |
| 10 | Presence of OA was according to valid definitions | CC/ CH | +/-/? |
| Study design |  |  |  |
| 11 | Prospective design was used | CH | +/-/? |
| 12 | Follow-up time ≥ 3 yr | CH | +/-/? |
| 13 | Withdrawals ≤ 20% | CH | +/-/? |
| 14 | Information on completers vs withdrawals | CH | +/-/? |
| Analysis and data presentation |  |  |  |
| 15 | Frequency of most important outcomes were given | CC/ CH | +/-/? |
| 16 | Appropriate analysis techniques were used | CC/ CH | +/-/? |
| 17 | Appropriate multivariate analysis techniques | CC/ CH | +/-/? |

CH: cohort study, CC: case-controlled study, +: positive response, -: inconsistent response, ?: unclear response.
