# Supplementary material for: Associations between weather conditions and osteoarthritis pain: a systematic review and meta-analysis
Source: Ann Med. 2023 Apr 20;55(1):2196439. doi: 10.1080/07853890.2023.2196439 (PMC10120534; doi:10.1080/07853890.2023.2196439)
Supplement: Supplemental Material [file IANN_A_2196439_SM6249.docx]

**The Medical Subject Headings (MeSH) and free text words of weather and OA**

**OA**: Osteoarthritis, Osteoarthritides, Osteoarthrosis, Osteoarthroses, Arthritis, Arthritides, Arthrosis, Arthroses, Osteoarthrosis Deformans.

**Weather:** Climate, Change Climate, Changes Climate, Climate Changes, Temperature, Meteorological, Factor Meteorological, Factors Meteorological, Weather, Extreme Weather, Wind, Wind Speed, Humidity, Cold Temperatures, Atmospheric Pressure, Atmospheres, Atmosphere, Precipitation，Sun, Sunshine, Rain, Rainy

**Search strategy**

**Pubmed**

**((("Weather"[Mesh]) OR ("Climate"[Mesh])) OR ((((((((((((((((((((Change Climate[Title/Abstract]) OR (Changes Climate[Title/Abstract])) OR (Climate Changes[Title/Abstract])) OR (Temperature[Title/Abstract])) OR (Meteorological[Title/Abstract])) OR (Factor Meteorological[Title/Abstract])) OR (Factors Meteorological[Title/Abstract])) OR (Extreme Weather[Title/Abstract])) OR (Wind[Title/Abstract])) OR (Wind Speed[Title/Abstract])) OR (Humidity[Title/Abstract])) OR (Cold Temperatures[Title/Abstract])) OR (Atmospheric Pressure[Title/Abstract])) OR (Atmospheres[Title/Abstract])) OR (Atmosphere[Title/Abstract])) OR (Precipitation[Title/Abstract])) OR (Sun[Title/Abstract])) OR (Sunshine[Title/Abstract])) OR (Rain[Title/Abstract])) OR (Rainy[Title/Abstract]))) AND ((("Osteoarthritis"[Mesh]) OR ((Osteoarthritides[Title/Abstract]) OR (Osteoarthrosis[Title/Abstract]))) OR ((((((Osteoarthroses[Title/Abstract]) OR (Arthritis[Title/Abstract])) OR (Arthritides[Title/Abstract])) OR (Arthrosis[Title/Abstract])) OR (Arthroses[Title/Abstract])) OR (Osteoarthrosis Deformans[Title/Abstract])))**

**Cochrane**

1 (Climate):ab,ti,kw OR (Temperature):ab,ti,kw OR (Wind):ab,ti,kw OR (Wind speed):ab,ti,kw OR (Humidity):ab,ti,kw OR (Weather):ab,ti,kw OR (Cold temperature):ab,ti,kw OR (Atmospheric pressure):ab,ti,kw OR (Change Climate):ab,ti,kw OR (Changes Climate):ab,ti,kw OR (Climate Changes):ab,ti,kw OR (Extreme Weather):ab,ti,kw OR (Meteorological):ab,ti,kw OR (Factor Meteorological):ab,ti,kw OR (Factors Meteorological):ab,ti,kw OR (Atmospheres):ab,ti,kw OR (Atmosphere):ab,ti,kw OR (Precipitation):ab,ti,kw OR (Sunshine):ab,ti,kw OR (Sun):ab,ti,kw OR (Rain):ab,ti,kw OR (Rainy):ab,ti,kw

2(Osteoarthrosis):ab,ti,kw OR (Osteoarthroses):ab,ti,kw OR (Arthritis):ab,ti,kw OR (Arthritides):ab,ti,kw OR (Arthrosis):ab,ti,kw OR (Arthroses):ab,ti,kw OR (Osteoarthrosis Deformans):ab,ti,kw

#1AND#2

**Web of Science**

1 TS=(Osteoarthritis OR Osteoarthritides OR Osteoarthrosis OR Osteoarthroses OR Arthritis OR Arthritides OR Arthrosis OR Arthroses OR Osteoarthrosis Deformans)

2 TS= (Climate OR Temperature OR Wind OR Wind speed OR Humidity OR Weather OR Cold Temperatures OR Atmospheric pressure OR Climate OR Change Climate OR Changes Climate OR Climate Changes OR Extreme Weather OR Atmospheric pressure OR Meteorological OR Factor Meteorological OR Factors Meteorological OR Meteorological OR Atmospheres OR Atmosphere OR Precipitation OR Sun OR Sunshine OR Rain OR Rainy)

3 (#1)AND#2

**Embase**

Query('Climate':ab,ti OR 'Temperature':ab,ti OR 'Wind':ab,ti OR 'Wind speed':ab,ti OR 'Humidity':ab,ti OR 'Weather':ab,ti OR 'Cold temperature':ab,ti OR 'Atmospheric pressure':ab,ti OR 'Change Climate':ab,ti OR 'Changes Climate':ab,ti OR 'Climate Changes':ab,ti OR 'Extreme Weather':ab,ti OR 'Factor Meteorological':ab,ti OR 'Meteorological':ab,ti OR 'Factors Meteorological':ab,ti OR 'Atmospheres':ab,ti OR 'Atmosphere':ab,ti OR 'Precipitation':ab,ti OR 'Sun':ab,ti OR'Sunshine':ab,ti OR'Rain':ab,ti OR'Rainy':ab,ti) AND ('Osteoarthritis':ab,ti 'Osteoarthritides':ab,ti OR 'Osteoarthrosis':ab,ti OR 'Osteoarthroses':ab,ti OR 'Arthritis':ab,ti OR 'Arthritides':ab,ti OR 'Arthrosis':ab,ti OR 'Arthroses':ab,ti OR 'Osteoarthrosis Deformans':ab,ti)
